# A Novel Mathematical Model for Studying Antimicrobial Interactions Against *Campylobacter jejuni*

**DOI:** 10.3389/fmicb.2019.01038

**Published:** 2019-05-14

**Authors:** Mohammed J. Hakeem, Khalid A. Asseri, Luyao Ma, Keng C. Chou, Michael E. Konkel, Xiaonan Lu

**Affiliations:** ^1^Food, Nutrition, and Health Program, Faculty of Land and Food Systems, The University of British Columbia, Vancouver, BC, Canada; ^2^Department of Food Science and Nutrition, College of Food and Agriculture Sciences, King Saud University, Riyadh, Saudi Arabia; ^3^Department of Anesthesiology, Pharmacology and Therapeutics, The University of British Columbia, Vancouver, BC, Canada; ^4^Department of Pharmacology, College of Pharmacy, King Khalid University, Abha, Saudi Arabia; ^5^Department of Chemistry, The University of British Columbia, Vancouver, BC, Canada; ^6^School of Molecular Biosciences, College of Veterinary Medicine, Washington State University, Pullman, WA, United States

**Keywords:** food safety, antimicrobials, encapsulation, FICI, isobologram, median-effect equation

## Abstract

The aim of this study is to investigate the antimicrobial synergistic effect against *Campylobacter jejuni*, a leading foodborne pathogen that causes human gastroenteritis, by cinnamon oil, encapsulated curcumin, and zinc oxide nanoparticles (ZnO NPs). We compared three approaches to study the antimicrobial interactions, including the time-killing method, the fractional inhibitory concentration index (FICI) method, and a mathematical concentration-effect model. Isobologram analysis was performed to evaluate the synergy in different combinations, and a median-effect equation was applied to identify the combinations of synergistic effects at median, bacteriostatic, and bactericidal reduction levels. The time-killing method overestimated the synergistic interaction between antimicrobials, while the FICI method failed to detect an existing synergistic phenomenon. This lack of accuracy and sensitivity was mainly due to combining antimicrobials without a deep understanding of their concentration-effect relationships. Our results showed that each antimicrobial had a unique concentration-effect curve. Specifically, encapsulated curcumin showed a sharp sigmoidal curve unlike cinnamon oil and ZnO NPs. A mathematical model was applied to study the interaction between antimicrobials with a different shape of concentration-effect curve. We observed an additive effect of cinnamon oil/ZnO NPs and synergistic interactions of other binary combinations (cinnamon oil/encapsulated curcumin and ZnO NPs/encapsulated curcumin). The tertiary combination of cinnamon oil/ZnO NPs/encapsulated curcumin at IC_25_ (additive line <1-log CFU/mL) presented the greatest synergistic effect by reducing the bacterial population over 8-log CFU/mL. This mathematical model provided an alternative strategy to develop a new antimicrobial strategy.

## Introduction

*Campylobacter* is one of the leading bacterial causes of human infectious diseases worldwide. In Canada, this microorganism causes ∼145,350 cases of foodborne illness per year (Thomas et al., 2013). *Campylobacter jejuni* is the most common species that accounts for ∼80% of campylobacteriosis with a relatively low infectious dose (∼500–800 cells) (Nachamkin et al., 2008). *C. jejuni* infections usually lead to non-fatal and self-limiting gastroenteritis, including watery diarrhea, nausea, and vomiting; however, severe autoimmune neurological disorders such as Guillain–Barré syndrome may occur in immunocompromised individuals (Kaakoush et al., 2015). Transmission of *C. jejuni* is commonly through the consumption of meat product (e.g., poultry and beef), raw milk, and/or contaminated drinking water. For all of these reasons, there is an urgent need to develop a new strategy of antimicrobial usage to reduce the prevalence of *Campylobacter* in the environment and agri-food products.

A combination of antimicrobials may target different bacterial sites and lead to synergistic interaction. This may form a novel and effective strategy of antimicrobial usage to reduce the prevalence of *Campylobacter* infections. Synergy is defined as an effect produced by two or more agents greater than the sum of their individual effects combined (i.e., additive effect) (Chou, 2006). Synergy requires a lower concentration of each agent to either increase or maintain the antimicrobial effect. Three methods have been used to study the synergism between antimicrobials, including disk diffusion, time-killing, and fractional inhibitory concentration index (FICI) methods (Odds, 2003; Zhou et al., 2016). Currently, there is no standard method for studying antimicrobial interactions. Over 60% of the dual antimicrobial studies used the FICI method and 36% applied the time-killing method during the past decade (Odds, 2003). Each method has advantages and disadvantages, and generates different outcomes that may not be comparable with each other (Rand et al., 1993; Odds, 2003; Chou, 2006; Noll et al., 2012; Zhou et al., 2016). For instance, the time-killing method investigates bactericidal effect over time, while the FICI method studies bacteriostatic effect after one time point (e.g., 24 h). Both methods are established on a linear concentration-effect curve of antimicrobials, which can result in either over or under-estimation of interaction(s). According to a recent report, only 40 out of 86 studies published between 1999 and 2015 used rigid mathematical methods to accurately study the synergetic effects of Chinese herbal medicine (Zhou et al., 2016). Although advanced pharmacological methods are widely used to study drug combination effect in the pharmaceutical and biomedical sciences, few of these studies are related to antimicrobials research (Odds, 2003; Mora-Navarro et al., 2015; Zhou et al., 2016).

The isobologram is commonly conducted in drug combination studies to identify and evaluate drug interactions. The combined drugs are assumed to be equally effective, but the dose-response of each combined drug is not always similar (Chou, 2006). In-depth studies have indicated that even if two drugs have the same effect at the reference concentration [e.g., minimum inhibitory concentration (MIC)], this equivalence may not occur at their sub-concentrations (Tallarida, 2001; Chou, 2006). Quantitative assessment is therefore essential to identify dose-response of individual drugs and distinguish these situations when the shapes of dose-response curves are not similar. Methods used for the study of drug interactions can be valid only if both drugs have hyperbolic dose-response curves. Drawing an accurate additive line based on every pair of concentrations can overcome this limitation regardless the assay used, shape of dose-response curve, and the level of interaction (Chou, 2006). Even if an interaction exists, not every pair of concentration results in the same level of interaction. Additional analysis involving the use of a median-effect equation can provide a more comprehensive understanding of interactions between antimicrobials.

The objective of the current study was to compare the time-killing method and FICI method with a mathematical model and provide an easy and accurate approach to study the effect of dual antimicrobials. We used a non-linear mathematical concentration-effect model to evaluate the synergistic interactions of three representative antimicrobials (i.e., cinnamon oil, encapsulated curcumin and ZnO NPs) against *C. jejuni* as a foodborne pathogen model. The advantage of using the mathematical model is not only to identify or evaluate the synergy but also to avoid some of the possible mathematical errors and increase the sensitivity to detect an existing synergistic interaction. To the best of our knowledge, this was the first study to apply a mathematical model that could accurately evaluate the antimicrobial interactions at median (50% reduction), bacteriostatic, and bactericidal levels. This approach can be generalized to quantitatively evaluate any type of dual antimicrobial treatment against microorganisms for different applications.

## Materials and Methods

### Chemicals and Reagents

Hydrophobically modified starch (HMS) HI-CAP^TM^ 100 was donated from Ingredion Canada Inc., (Mississauga, ON, Canada). Cinnamon oil and curcumin purified from turmeric powder were purchased from Sigma-Aldrich (Oakville, ON, Canada). A powdered form of ZnO NPs (size: 40–100 nm, surface area: 12–24 m^2^/g) was obtained from Alfa Aesar (Haverhill, MA, United States). Acetic acid, acetonitrile, chloroform and dimethyl sulfoxide (DMSO) were purchased from Sigma-Aldrich (Oakville, ON, Canada).

### Preparation, Extraction and Quantification of Encapsulated Curcumin

To increase the water solubility, curcumin was encapsulated by HMS according to the protocol described in a previous study (Yu and Huang, 2010). Briefly, 1.8 g of curcumin was added into 1% (w/v) HMS solution, homogenized at 8,000 rpm for 10 min by using Omni Mixer Homogenizer (Omni, Kennesaw, GA, United States), followed by stirring for 24 h at room temperature. The suspension was centrifuged at 11,617 × *g* for 5 min. Supernatant was collected and filtered through a 0.45-μm nylon syringe membrane (Milliporesigma, Mississauga, ON, United States). Filtered encapsulated curcumin was placed at −80°C for 4 h and then freeze-dried at −53°C using a 12-L Console Labconco freeze-dryer (Kansas city, MO, United States) for 24 h. The freeze-dried product was packaged and stored at −20°C for further use. The negative control was that of HMS processed without the addition of curcumin.

Encapsulated curcumin was extracted and quantified as follows. First, 1.5 mL of double-deionized water was added to 0.5 g of freeze-dried encapsulated curcumin. The curcumin aqueous solution was then mixed with an equal volume of chloroform in a 10-mL glass tube. Two-phase liquid-liquid emulsions were obtained and then stirred at 200 rpm for 10 min to remove any free curcumin. Next, the emulsion was mixed with chloroform at the same volume, followed by vortex for 10 min and stirring at 200 rpm for overnight. Chloroform paste was collected then filtered through a 0.45-μm membrane before quantification. The extracted curcumin was analyzed using an Agilent 1260 HPLC system coupled with a diode array detector (HPLC-DAD; Agilent Technology, Santa Clara, CA, United States). An aliquot (20 μL) of sample was injected into a SUPELCOSIL^TM^ C_18_ column (300 × 4.6 mm, 3 μm; Sigma-Aldrich, Oakville, ON, Canada) and eluted at a flow rate of 1.0 mL/min and ambient temperature. The extracted curcumin was analyzed using an Agilent 1260 HPLC system coupled with a diode array detector (HPLC-DAD; Agilent Technology, Santa Clara, CA, United States). An aliquot (20 μL) of sample was injected into a SUPELCOSIL^TM^ C_18_ column (300 × 4.6 mm, 3 μm; Sigma-Aldrich, Oakville, ON, Canada) according to the protocol described in a previous study (Jayaprakasha et al., 2002). The mobile phase consisted of (A) 2% acetic acid (v/v) in water and (B) acetonitrile. A linear gradient was conducted from 45% B to 65% B within 8 min, then to 90% B before 10 min, and returned to 45% B before 15 min. Chromatograms were obtained at 425 nm. The retention time of curcumin was 7.5 min.

### Bacterial Strains

The *C. jejuni* F38011 (clinical isolate), human10 (clinical isolate) (Li et al., 2017), and ATCC 33560 (bovine feces isolate) strains were routinely cultivated either on *Campylobacter* agar (OXOID, Nepean, ON, Canada) plates supplemented with 5% defibrinated sheep blood (Alere Inc., Stittsville, ON, Canada) for 48 h or in Mueller–Hinton (MH) broth with constant shaking for 18 h at 37°C in a microaerobic environment (i.e., 10% CO_2_). Overnight *C. jejuni* cultures were individually prepared to 1 × 10^9^ CFU/mL by adjusting OD_600_ value. Then, a cocktail culture was prepared by combining equal volumes of each of the three cultures for further antimicrobial testing.

### Investigation of Synergistic Antimicrobial Effect Using Time-Killing Method

The minimum bactericidal concentrations (MBC) of cinnamon oil, ZnO NPs, and encapsulated curcumin against *C. jejuni* were separately determined using time-killing method (Wiegand et al., 2008). A series of two-fold dilutions from 1 to 100 ppm of antimicrobials were individually mixed with *C. jejuni* cocktail, with the initial cell count of 10^5^ CFU/mL (Supplementary Table S1), and the survival cells were determined after different time intervals using the conventional platting assay. The MBC value was used as the reference concentration for synergistic antimicrobial testing. Different concentrations (2×, 1×, 0.5× and 0.25× MBC) of cinnamon oil were individually combined with the MBC of ZnO NPs and *C. jejuni* cocktail culture (Supplementary Table S1). To identify the interaction between antimicrobials, the results of individual antimicrobial treatments were compared to that of the combined antimicrobial treatment. Log reduction was calculated by subtracting the bacterial count in treated group from the control group at the same time point. The experiment was conducted in triplicates.

### Investigation of Synergistic Antimicrobial Effect Using Fractional Inhibitory Concentration Index Method

The interaction of antimicrobial effects between cinnamon oil, encapsulated curcumin, and ZnO NPs was also evaluated using the FICI method. The minimum bacteriostatic concentration (MIC) of single and dual antimicrobials was determined then the FICI of each combination was identified as follows: FICI = (MIC of A in combination / MIC of A alone) + (MIC of B in combination / MIC of B alone), where A and B represent a different antimicrobial agent. Tests were performed at FICI values of 0.3, 0.4, 0.5, and 1 in order to identify the type of interactions between antimicrobials. The minimum FICI value that inhibited bacterial growth was defined as synergistic (≤0.5), additive (0.5-4), or antagonistic (>4) (Odds, 2003). Antimicrobials were prepared in fresh MH medium containing *C. jejuni* cocktail with an initial cell density of 10^5^ CFU/mL. The procedure was, similarly applied in all standardized susceptibility methods (Wiegand et al., 2008). The inhibitory effect was reported as either positive or negative based upon the clarity (+) or turbidity (−) of the tested bacterial cultures. The experiment was conducted in triplicates.

### Investigation of Synergistic Antimicrobial Effect Using Mathematical Modeling

#### Concentration-Effect Curves of Single Antimicrobials

A broad range of concentrations of cinnamon oil, ZnO NPs, and encapsulated curcumin were tested against *C. jejuni* strain F38011 to identify the concentration-effect curve after a 3 h of treatment. Each antimicrobial was prepared at a series of concentrations (Supplementary Table S2) and then individually mixed with *C. jejuni* at an initial concentration of 10^8^ CFU/mL, followed by incubation at 37°C for 3 h in a microaerobic environment. Antimicrobial testing was performed using the conventional plating assay. The experiment was conducted in triplicates. Antimicrobial effect was reported as a percent and log reduction of the bacterial cells. Concentration-effect curves were generated using Prism 5 software (GraphPad, San Diego, CA, United States).

#### Preparation of Antimicrobial Combinations

Concentration-effect data were used to prepare different pairs of concentrations based on their potencies (e.g., IC_20_ + IC_20_). The inhibitory concentrations (e.g., IC_20_) used for preparing pairs of concentrations were determined by:

(1) $${\mathrm{IC}}_{F}={\left(\frac{F}{100-F}\right)}^{\frac{1}{H}}\times {\mathrm{IC}}_{50}$$

where *IC*_F_ represents the inhibitory concentration (e.g., IC_20_), *F* denotes the cell percentage reduction (e.g., 20%), *H* stands for the Hill slope, and IC_50_ is the inhibitory concentration that gives 50% reduction of the cells. The theoretical additive effect (e.g., the sum effect of IC_20_ + IC_20_) was calculated by the fractional product method (Chou and Talalay, 1984):

$$(1-f_{1})(1-f_{2})=V_{1\&2}$$

Theoretical additive effect

(2) $$(\%)=(1-V_{1\&2})\times 100$$

where *f* represents the fraction of cell reduction (CFU/mL) by single antimicrobials (i.e., *f*_1_ and *f*_2_) and *V*_1&2_ represents the theoretical fractional concentration of viable cells after treatment with two antimicrobials. Combined effect can be synergistic, additive, or antagonistic. Synergistic effect takes place when the combined effect is greater than the theoretical additive effect, while additive or antagonistic effects take place when the combined effect is equal to or lower than the theoretical additive effect, respectively. The maximum theoretical additive effect used in this study was 82% (<1 log) CFU/mL to ensure that the antimicrobial combinations do not completely inactivate the entire bacterial population. In this case, the 18% remaining population accounts for (>7 log) CFU/mL.

#### Isobologram

Isobologram analysis was used to investigate the interaction effect (synergistic, antagonistic, or additive interaction) between different binary combinations. Sets of equally effective concentrations were selected to generate isobolograms according to the method described in a previous study (Chou and Talalay, 1984). The IC_50_ of each single antimicrobial was used to draw an additive line between the combined antimicrobials. Reduction of antimicrobials was calculated according to:

(3) R=IC50 in combinationIC50 alone×100

where *R* represents the reduction of antimicrobial concentration. The isobolograms were generated using Prism 5.

#### Median-Effect Plot

A systematic analysis of the concentration-effect data of single and combined antimicrobials was conducted to generate a median effect plot. The data were normalized by Chou’s median effect equation (Chou, 2006) as follows:

(4) $$\mathrm{Log}\frac{F}{100-F}=\mathrm{Log}{\left(\frac{C}{{\mathrm{IC}}_{50}}\right)}^{H}$$

where *C* represents the concentration of antimicrobial(s). The median effect plot was generated using Prism 5.

### Statistical Analysis

Prism software (version 5.01: GraphPad Software Inc., San Diego, CA, United States) was used for statistical analysis and the graphs generation. The time-killing data were analyzed by one-way ANOVA, followed by *post hoc* Tukey’s test for multiple comparisons. A *P* value was adjusted at 0.05 or less to define statistically significant differences between and within groups.

## Results and Discussion

### Conventional Methods Used to Study the Antimicrobial Interactions

#### Time-Killing Method

**FIGURE 1:**
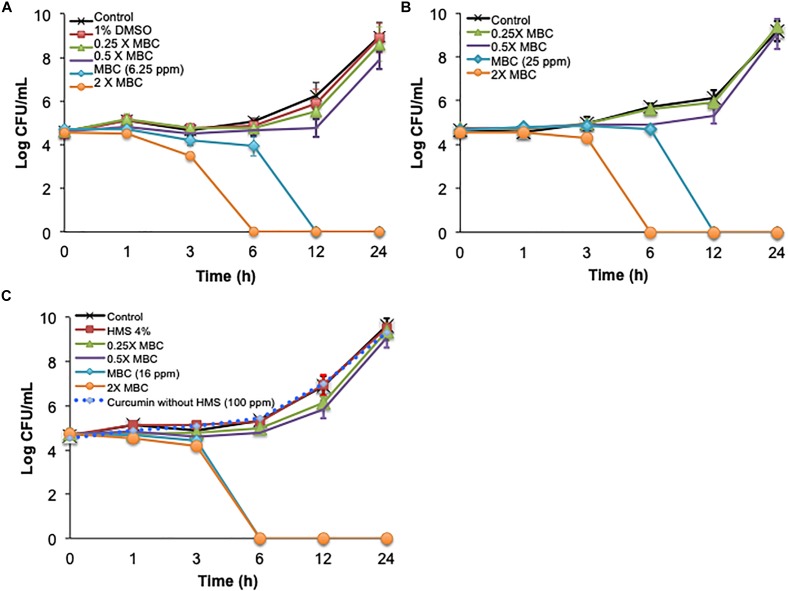
Time-killing curves of *C. jejuni* cocktail with single antimicrobial treatment at 37°C in a microaerobic condition. The antimicrobial effect of **(A)** cinnamon oil, **(B)** zinc oxide nanoparticle (ZnO NP), and **(C)** encapsulated curcumin against C *jejuni* is summarized. Control represents the untreated cells, MBC is the minimum bactericidal concentration, and HMS reflects hydrophobically modified starch. Error bars represent the standard deviation (*n* = 3, duplicates).

**FIGURE 2:**
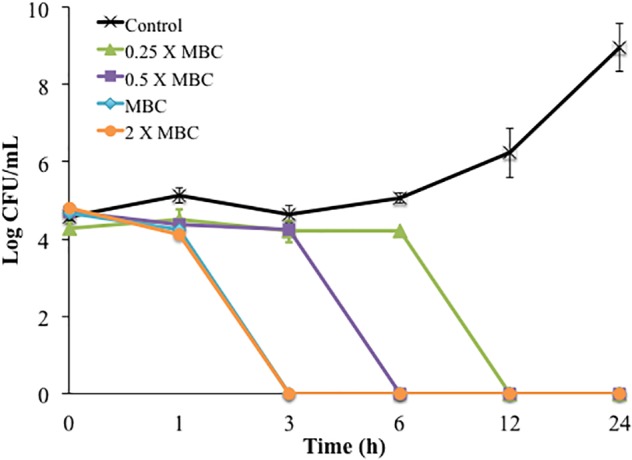
Time-killing curves of *C. jejuni* cocktail treated with different concentrations of cinnamon oil (0.25 – 2 X MBC) combined with 0.5 X MBC of ZnO NP (MBC = 25 ppm) at 37°C in a microaerobic condition. Control represents the untreated cells. MBC is the minimum bactericidal concentration. Error bars represent the standard deviation (*n* = 3, duplicates).

The time-killing method was used first to study single and combined antimicrobial treatments. Cinnamon oil, ZnO NPs, and encapsulated curcumin showed bactericidal activity at 1 × MBC and 2 × MBC after 3, 6, 12, and 24 h (*P* < 0.0001) (Figure 1A). The 0.5 × MBCs of all single antimicrobials had a mild effect with ≤1 log reduction and no significant effect compared to the control groups at all time points (*P* > 0.05) (Figure 1). The combination of cinnamon oil and ZnO NPs at low concentrations (≤0.5 × MBC) significantly enhanced the antimicrobial effect. For example, ZnO NPs at 0.5 × MBC and cinnamon oil at 0.25 × MBC resulted in a 6.24 log reduction of *C. jejuni* after 12 h of treatment (*P* < 0.0001) (Figure 2), while the same concentrations of both single antimicrobials showed no significant difference (*P* > 0.05) compared to the control group even after 24 h (Figure 1A,B).

Dimethyl sulfoxide (1%) did not significantly affect cell viability (*P* > 0.05) (Figure 1A), indicating that the antimicrobial effect of cinnamon oil solution was not due to the solvent. Similarly, up to 100 ppm of free curcumin (without encapsulation or HMS) and 1% HMS had no effect on the viability of *C. jejuni* (*P* > 0.05) (Figure 1C). The effects of 1% DMSO or 1% HMS with and without antimicrobial agents (4 × MIC) were significantly different after 3, 6, 12, and 24 h (*P* < 0.0007) indicating no interaction between these compounds and antimicrobials against cell viability (Figure 1A,C).

Synergism was identified when two antimicrobials resulted in a greater log reduction than the sum of their individual effects [34–36]. In the current study, the single treatment of cinnamon oil (0.5 × MBC) or ZnO NPs (0.5 × MBC) induced a ≤1 log reduction after 24 h (*P* > 0.05) (Figure 1A,B). An additive effect was considered if the combined treatment caused ∼2 log reduction after 24 h. Interestingly, the combination of cinnamon oil (0.5 × MBC)/ZnO NPs (1 × MIC) resulted in 5 log reduction within 6 h of treatment (*P* < 0.0001) (Figure 2), inducing additional log-reduction (CFU/mL) compared to the additive effect. Therefore, cinnamon oil and ZnO NPs were considered to have a synergistic effect against *C. jejuni*. Although previous studies used this approach to study antimicrobial synergic effect (Ghosh et al., 2013; Ha and Kang, 2015; Huq et al., 2015), it measures the effect on the basis of logarithmic scale, leading to potential overestimation of the interaction between the two antimicrobials. For example, the 0.5 × MBC of all single antimicrobials showed ≤1 log reduction (≤90% of bacterial population) (Figure 1, 2). If 90% of bacterial cells can be inactivated by one antimicrobial at the 0.5 × MBC, it is likely that the other antimicrobial can easily inactivate the remaining population (∼10% of the viable cells) at its 0.5 × MBC when both antimicrobials are applied at the same time. It is not reasonable to draw an additive line for two antimicrobials based on their log reductions because the sum of the two combined concentrations may fully inactivate the entire bacterial population. Although the time-killing method has many advantages including that the antimicrobial effects are monitored over time, our current results showed that this method overestimated the synergism between cinnamon oil and ZnO NPs. Thus, other methods were further applied to investigate the synergism between the selected antimicrobials.

#### FICI Method

The FICI method has been frequently used to study antimicrobial interactions because it is simple and can test many concentrations simultaneously (Odds, 2003). The FICI method relies on combining multiple sub-MICs. The MIC at specific FICI value indicates the type of interaction. The additive effect takes place when the FICI is >0.5-1 (Odds, 2003). A lower FICI value indicates a higher synergism while a higher FICI value indicates antagonism. We used the FICI method to study the interactions between the binary combinations of cinnamon oil/ZnO NPs, cinnamon oil/encapsulated curcumin, and ZnO NPs/encapsulated curcumin. *C. jejuni* cocktails (5 log CFU/mL) were treated with antimicrobials for 24 h. All of the combinations did not show any inhibitory effect at a FICI value of 0.3, 0.4, or 0.5 (Table 1), indicating that no synergistic interaction occurred. In comparison, all combinations inhibited the growth of *C. jejuni* at a FICI value of 1, demonstrating an additive effect. Unlike the time-killing method, the FICI method did not show any synergistic interactions between tested antimicrobials (Figure 2 and Table 1).

**Table 1:** Antimicrobial effect of binary combinations of cinnamon oil, zinc oxide nanoparticle and encapsulated curcumin on *C. jejuni* cocktail.

FICI^∗^	Fraction of MIC		A&B	Cinnamon oil & ZnO NPs	Cinnamon oil & Encapsulated curcumin	ZnO NPs & Encapsulated curcumin
	A	B				
0	0	0		+	+	+
1	0.5	0.5		−	−	−
1	0.25	0.75		−	−	−
1	0.75	0.25		−	−	−
0.5	0.25	0.25		+	+	+
0.4	0.2	0.2		+	+	+
0.3	0.15	0.15		+	+	+

*The turbidity of the bacterial culture was observed after 24 h of incubation at 37°C in a microaerobic condition. Initial bacterial count was 10^5^ CFU/mL. The “+” denotes turbid and the “–” indicates clear (n = 3, duplicates). The average bacterial concentration in the turbid samples was 9.69 ± 0.27 log CFU/mL. ^∗^FICI: fractional inhibitory concentration index.*

**FIGURE 3:**
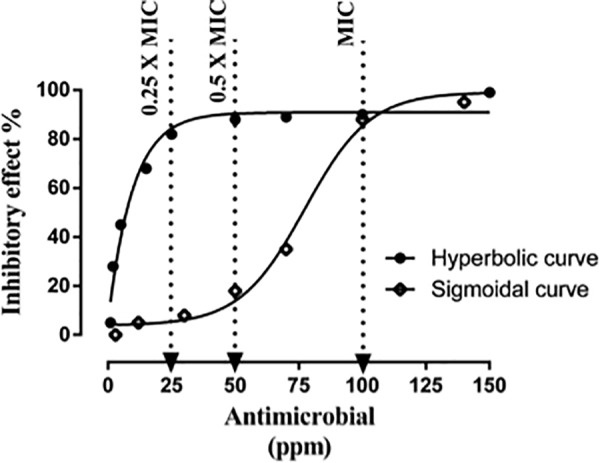
Predicted example of combining two antimicrobials based on their sub-MICs. Inhibitory effect represents the percentage of inactivated cells. Increasing the concentration in the hyperbolic curve from 50 to 100 ppm raises the inhibitory effect by 5% (from 85 to 90%). Increasing the concentration in the sigmoidal curve from 50 to 100 ppm raises the inhibitory effect by more than 3-fold (from 18 to 88%).

Both the time-killing method and the FICI method rely on the assumption that antimicrobials are equally effective at MICs/sub-MICs or MBC/sub-MBC. However, even when two antimicrobials at MICs cause similar effects, the antimicrobial effect at their sub-MICs might be totally different from each other depending on the shape of their concentration-effect curve [37]. It is difficult to predict the additive effect by combining two antimicrobials with different concentration-effect shapes. The extreme difference between two antimicrobials in their concentration-effect relationship highlights the challenge to predict the additive line of antimicrobial combinations in the conventional methods. Moreover, the potency of each antimicrobial can be unequal when they are mixed at 0.50 × or 0.25 × MICs (Figure 3). Friedman et al. (2002) tested the antimicrobial effects of 96 essential oils and 23 purified oil compounds against *C. jejuni*, *Escherichia coli*, *Listeria monocytogenes*, and *Salmonella enterica* and obtained different shapes of concentration-effect curve. Although this is a common problem, none of the conventional methods take this into consideration. Our results (Table 1) agreed with a previous study demonstrating the inability of FICI method to identify synergism between synergistic antimicrobials (Lambert and Lambert, 2003). The limitation is simply due to different dose-response of the combined antimicrobials. Therefore, it is important to use alternative methods to discover new synergistic combinations that are not detected by the conventional methods. Taken together, FICI may not be able to identify an existing synergism between antimicrobials if they have different concentration-effect curves.

### Mathematical Modeling Used to Study the Antimicrobial Interactions

A non-linear mathematical concentration-effect model was used to combine antimicrobials in a more detailed manner using a selected strain and a selected time point. The isobologram and median-effect equation were used to analyze the data (Chou and Talalay, 1984; Tallarida, 2001; Chou, 2006; Tallarida, 2006). The idea was to combine antimicrobials based on their quantitative potencies (i.e., IC_40_ + IC_40_) instead of sub-MICs or sub-MBCs. Potency is the antimicrobial effect of a specific concentration (e.g., IC_50_) of an antimicrobial agent, while efficacy it the maximum effect induced by an antimicrobial. *C. jejuni* strain F38011 was selected because it was isolated from an individual with bloody diarrhea (O’Loughlin et al., 2015), effectively colonizes the chicken gastrointestinal tract (Konkel et al., 1998), and causes illness in mice and pigs (O’Loughlin et al., 2015). Only a single time point (i.e., 3 h) was selected to study the concentration-effect curve for each antimicrobial because 3 h was identified to be the minimum time period to cause *Campylobacter* reduction by single or dual antimicrobials (Figures 1, 2). Similarly, another study tested the antimicrobial effect of ZnO NPs and identified that 3 h of treatment was the minimum time to reduce the count of *C. jejuni* (Xie et al., 2011). Taken together, different steps and considerations were used in a non-linear concentration-effect mathematical model to study the antimicrobial interactions.

#### Concentration-Effect Curves

**FIGURE 4:**
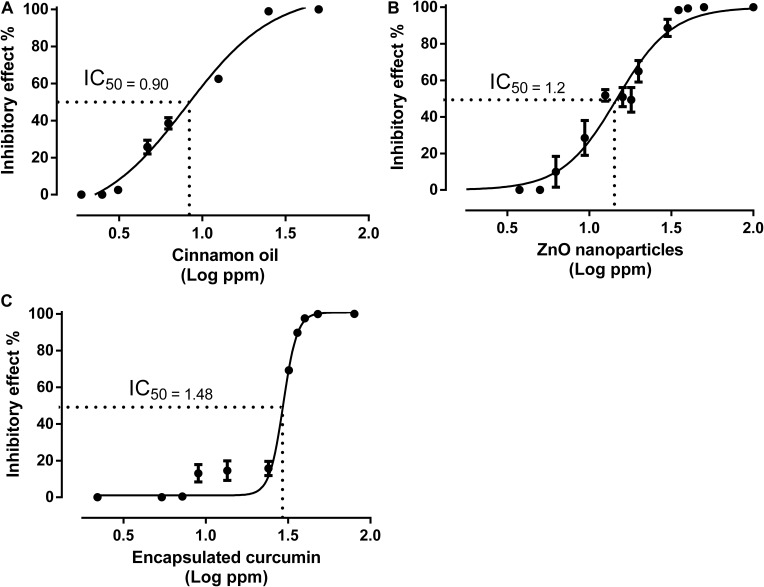
Concentration-effect curves for cinnamon oil **(A)**, ZnO NP **(B)**, and encapsulated curcumin **(C)** against *C. jejuni* strain F38011 after 3 h treatment at 37°C in a microaerobic condition. Inhibitory effect represents the percentage of inactivated cells, Error bars represent the standard deviation (*n* = 3, duplicates).

All antimicrobials demonstrated a non-linear regression between the concentrations and antimicrobial effects (Figure 4). Cinnamon oil and ZnO NPs showed a similar shape of concentration-effect curve with a slight difference, while encapsulated curcumin presented a sharp sigmoidal curve. IC_50_ is a representative concentration used to evaluate the potency of drugs or antimicrobials. The lower the IC_50_ the more potent is the drug. Up to thirteen concentration-effect points were used to generate high quality concentration-effect curves. Some points showed no effect; some were on the slope; and some were on the plateau of their concentration-effect curves. Other methods, such as the FICI, rely on one reference concentration (MIC) for studying antimicrobial interactions. We used multiple data points and identify the IC_50_ as a representative concentration (located on the middle of the concentration-effect slope) to determine nine other reference concentrations. The IC_50_ of cinnamon oil, ZnO NPs, and encapsulated curcumin were identified to be 0.90, 1.20, and 1.48 log ppm, respectively (Figure 4). All three individual antimicrobials had the same efficacy because they were all able to reduce bacterial population over 99% at 1.39 log ppm for cinnamon oil, 1.54 log ppm for ZnO NPs, and 1.60 log ppm for encapsulated curcumin (Figure 4).

#### Isobologram

**FIGURE 5:**
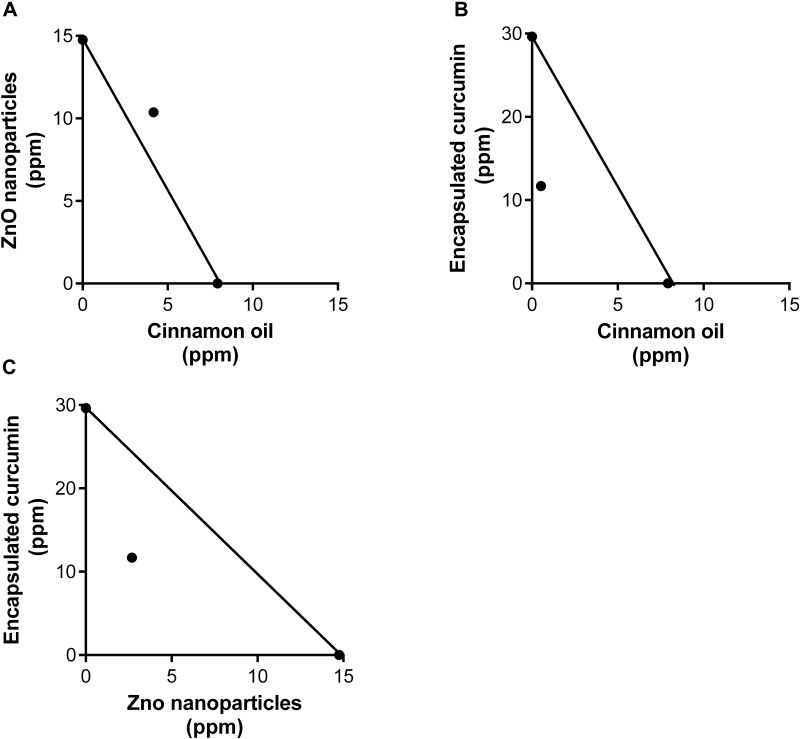
Isobologram analysis of binary combinations of cinnamon oil/ZnO NPs **(A)**, cinnamon oil/encapsulated curcumin **(B)**, ZnO NP/encapsulated curcumin **(C)** against *C. jejuni* strain F380I1 after 3 h treatment at 37°C in a microaerobic condition. The additive lines connect the IC_50_ of each single antimicrobial. Axial points show the IC_50_ of antimicrobial combinations. An axial point on the additive line indicates additive interaction. An axial point under the additive line indicates synergistic interaction (*n* = 3, duplicates).

The isobologram is a diagram that is commonly used to identify the type of interaction of a combination by comparing the concentrations of two single agents (*x* and *y*-axis) and their combination (axial point) (Figure 5). The IC_50_ of single antimicrobial cinnamon oil (7.92 ppm), ZnO NPs (14.75 ppm) and encapsulated curcumin (29.63 ppm) were used to draw additive lines. The additive effect is indicated if the combined (IC_50_) data point is on the additive line and synergism is indicated if the combined IC_50_ data point is below the additive line. The combination of cinnamon oil/ZnO NPs (4.14 + 10.37 ppm) indicated an additive interaction (Figure 5A). In contrast, cinnamon oil/encapsulated curcumin (0.523 + 11.70 ppm) (Figure 5B) and encapsulated curcumin/ZnO NPs (11.70 + 4.14 ppm) (Figure 5C) indicated synergistic interaction. Synergism between cinnamon oil and encapsulated curcumin resulted in the reduction of antimicrobial concentrations by 93.40% and 60.51%, respectively (Figure 5B). In comparison, ZnO NPs and encapsulated curcumin were reduced by 81.76% and 60.51%, respectively (Figure 5C). Taken together, up to 93.40% of the antimicrobial concentrations were reduced due to the synergistic interactions.

#### Median-Effect Curves

The median-effect principle has been employed to analyze the dose-response data in enzymatic, cellular, and animal studies. A few studies have used this approach to study the antimicrobial effects, such as the ones of using the median-effect equation to investigate the antiviral synergistic effect against human immunodeficiency virus (HIV) and influenza virus A (Asin-Milan et al., 2014; Kulkarni et al., 2014; Belardo et al., 2015). Most studies used the CompuSyn software to generate the median-effect plot (Chou, 2010). However, this software does not consider the shape of dose-response. Hence, it cannot overcome some of the possible mathematical errors in combinational studies. In the current study, we used non-linear concentrations-effect data to generate the median-effect plot and identified the overall effect of antimicrobials and their combinations in a wide range of concentrations. By using the median-effect principle, more meaningful data were provided because the IC_50_ shown in the isobologram was not sufficient for treatments (Figure 5).

**FIGURE 6:**
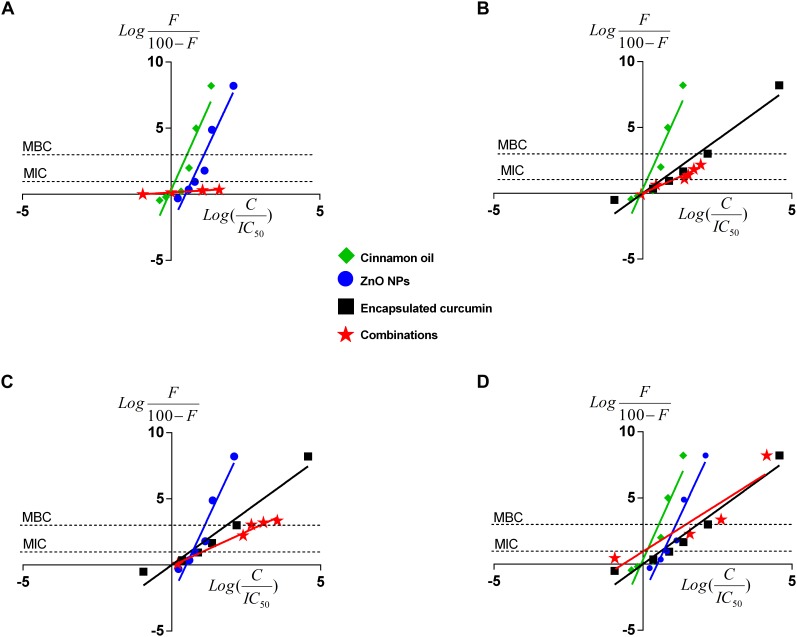
Median-effect plots of cinnamon oil, ZnO NP, and encapsulated curcumin against *C. jejuni* F38011 strain after 3-h treatment at 37°C in a microaerobic condition. Data were plotted for single or dual treatments as binary **(A–C)** or tertiary combinations **(D)**. *F* represents the percentage reduction of bacterial cells. *C* means the antimicrobial concentration and *H* is the slope hill of concentration-effect curves. Each line represents the effect of single or combinational antimicrobial treatment. The “0” on the *x*-axis represents median-effect and the “0” on the *y*-axis represents a 50% reduction of viable cells. The two unconnected lines show the antimicrobial effects at MIC (90% reduction) and MBC (99.9% reduction) of viable cells. The line slope represents the potency of the antimicrobial treatment(*n* = 3, duplicates).

Figure 6 shows the median-effect curves of single antimicrobials and their combinations at a broad range of concentration-effect relationship between 0 and >8 log reduction of bacterial cells. Cinnamon oil was identified to be the most potent antimicrobial because its median-effect curve reached to the maximum effect (>8 log) first followed by ZnO NPs, and then the encapsulated curcumin (Figure 6). Although we did not study the mode of action of single or dual antimicrobials, the median-effect curves of cinnamon oil and ZnO NPs had the same slope, suggesting that they might work with a similar mechanism (Figure 6A). The primary antibacterial functions of cinnamon oil and ZnO NPs are both related to the disruption of bacterial cell wall and cell membrane (Davidson et al., 2005; Xie et al., 2011). In contrast, curcumin acts as a cytokinesis inhibitor by direct interaction with FtsZ (Rai et al., 2008), an essential bacterial cell-division protein. This might explain why the encapsulated curcumin showed a sharp sigmoidal concentration-effect curve (Figure 4) and a very steep median-effect curve (Figure 6B–D).

Several pairs of the fixed ratio concentrations (from IC_10_ to IC_40_) were used to generate the median-effect plot. The median-effect line of cinnamon oil/ZnO NPs was almost horizontal and unable to cross the MIC (i.e., additive) line (Figure 6A and Table 2A). In comparison, all other combinations showed synergistic interactions and were able to cause antimicrobial reduction about three times greater (3 log-reduction) than the additive line (>1 log reduction) (Figure 6B,C and Tables 2B,C). The tertiary combination of cinnamon oil/ZnO NPs/encapsulated curcumin had the greatest synergistic interaction among the tested combinations because mixing three concentrations of IC_25_ (additive line 57%) resulted in over 8 log reduction of bacterial viable cells (Figure 6D and Table 2D). The median-effect curves of all-synergistic combinations were parallel with the curve of the encapsulated curcumin (Figure 6B,C), suggesting that encapsulated curcumin played an important role in the synergistic interaction.

**Table 2:** Binary (A–C) and tertiary (D) combinations of cinnamon oil, encapsulated curcumin, and ZnO NP against *C. jejuni* F38011 after 3 h treatment at 37°C in microaerobic condition (*n* = 3, duplicates).

A	Cinnamon oil potency (ppm)	ZnO NPs potency (ppm)		Additive line %	Effect %	Interaction	Log reduction	*SD*
	IC_10_ (2.16)	IC_10_ (6.78)		19	0.00	Additive	0.00	0.124
	IC_15_ (2.84)	IC_15_ (8.11)		27	26.99	Additive	0.14	0.027
	IC_25_ (4.14)	IC_25_ (10.37)		43	50.00	Additive	0.30	0.205
	IC_40_ (6.24)	IC_40_ (13.55)		64	55.42	Additive	0.35	0.020
	IC_30_ (4.81)	IC_60_ (18.53)		72	48.57	Additive	0.29	0.076
	IC_60_ (10.09)	IC_30_ (11.42)		72	45.09	Additive	0.26	0.255
	IC_10_ (2.16)	IC_80_ (27.05)		82	56.44	Additive	0.36	0.128
	IC_80_ (18.04)	IC_10_ (6.78)		82	72.80	Additive	0.57	0.068

**B**	**Cinnamon oil potency (ppm)**	**Enc. curcumin potency (ppm)**		**Additive line %**	**Effect %**	**Interaction**	**Log reduction**	***SD***

	IC_1_ (0.523)	IC_1_ (11.70)		2	44.58	Synergistic	0.26	0.187
	IC_5_ (1.38)	IC_5_ (14.38)		9	81.64	Synergistic	0.74	0.573
	IC_10_ (2.16)	IC_10_ (22.97)		19	92.56	Synergistic	1.13	0.012
	IC_15_ (2.84)	IC_15_ (24.02)		27	95.98	Synergistic	1.40	0.052
	IC_25_ (4.14)	IC_25_ (25.53)		43	98.53	Synergistic	1.83	0.523
	IC_40_ (6.24)	IC_40_ (27.29)		64	99.34	Synergistic	2.18	0.462
	IC_30_ (4.81)	IC_60_ (29.51)		72	99.42	Synergistic	2.24	0.264
	IC_60_ (10.09)	IC_30_ (26.16)		72	99.63	Synergistic	2.43	0.012
	IC_10_ (2.16)	IC_80_ (32.43)		82	99.99	Synergistic	4.45	0.168
	IC_80_ (18.04)	IC_10_ (22.97)		82	99.95	Synergistic	3.29	0.048

**C**	**Enc. curcumin potency (ppm)**	**ZnO NPs potency (ppm)**		**Additive line %**	**Effect %**	**Interaction**	**Log reduction**	***SD***

	IC_1_ (11.70)	IC_1_ (2.69)		2	49.08	Synergistic	0.29	0.071
	IC_5_ (14.38)	IC_5_ (5.80)		9	54.91	Synergistic	0.35	0.552
	IC_10_ (22.97)	IC_10_ (6.78)		19	99.42	Synergistic	2.24	0.050
	IC_15_ (24.02)	IC_15_ (8.11)		27	99.91	Synergistic	3.05	0.419
	IC_25_ (25.53)	IC_25_ (10.37)		43	99.94	Synergistic	3.21	0.059
	IC_40_ (27.29)	IC_40_ (13.55)		64	99.96	Synergistic	3.35	0.019
	IC_30_ (26.16)	IC_60_ (18.53)		72	99.99	Synergistic	8.21	0
	IC_60_ (29.51)	IC_30_ (11.42)		72	99.99	Synergistic	8.21	0
	IC_10_ (22.97)	IC_80_ (27.05)		82	99.99	Synergistic	5.12	0
	IC_80_ (32.43)	IC_10_ (6.78)		82	99.99	Synergistic	8.21	0

**D**	**Cinnamon oil potency (ppm)**	**ZnO NPs potency (ppm)**	**Enc. curcumin potency (ppm)**	**Additive line %**	**Effect %**	**Interaction**	**Log reduction**	***SD***

	IC_1_ (0.523)	IC_1_ (2.69)	IC_1_ (11.70)	3	50.00	Synergistic	0.21	0.031
	IC_5_ (1.38)	IC_5_ (5.80)	IC_5_ (14.38)	14	55.06	Synergistic	0.35	0.026
	IC_10_ (2.16)	IC_10_ (6.78)	IC_10_ (22.97)	27	99.49	Synergistic	2.29	0.002
	IC_25_ (4.14)	IC_25_ (10.37)	IC_10_ (22.97)	50	99.66	Synergistic	2.46	0.093
	IC_15_ (2.84)	IC_15_ (8.11)	IC_15_ (24.02)	43	99.96	Synergistic	3.36	0.150
	IC_25_ (4.14)	IC_10_ (6.79)	IC_25_ (25.53)	50	99.99	Synergistic	8.21	0
	IC_10_ (2.16)	IC_25_ (10.37)	IC_25_ (25.53)	50	99.99	Synergistic	8.21	0
	IC_25_ (4.14)	IC_25_ (10.37)	IC_25_ (25.53)	57	99.99	Synergistic	8.21	0

## Conclusion

In conclusion, conventional methods either overestimated or failed to detect the exiting synergic antimicrobial interactions due to the undefined concentration-effect curves. We were able to identify reference concentrations and evaluate the synergism even between antimicrobials with different concentration-effect curves. Up to 93.40% of the antimicrobial concentrations were reduced while maintaining the same effect due to synergistic interactions. Cinnamon oil and ZnO NPs acted differently from the encapsulated curcumin. We propose that this mathematical modeling can aid in developing new synergistic combinations to potentially reduce the prevalence and survival of foodborne pathogens as well as open the door to discover new mechanisms of dual antimicrobials.

## Author Contributions

MH provided the idea of the study, conducted the experiments, and wrote the manuscript. KA contributed to the idea of the study and analyzed the data. LM reviewed and edited the manuscript. KC reviewed and helped the mathematical modeling. MK reviewed and edited the manuscript. XL supervised, reviewed, and edited the manuscript.

## Conflict of Interest Statement

The authors declare that the research was conducted in the absence of any commercial or financial relationships that could be construed as a potential conflict of interest.
