# Supplementary material for: A Novel Mathematical Model for Studying Antimicrobial Interactions Against Campylobacter jejuni
Source: Front Microbiol. 2019 May 14;10:1038. doi: 10.3389/fmicb.2019.01038 (PMC6527739; doi:10.3389/fmicb.2019.01038)
Supplement: Supplementary file 1 [file Table_1.DOCX]

A novel mathematical model for studying antimicrobial interactions against *Campylobacter jejuni*

Mohammed J. Hakeem^1,2^, Khalid A. Asseri ^3,4^, Luyao Ma ^1^, Keng C. Chou ^5^, Michael E. Konkel ^6^, Xiaonan Lu ^1*^

| Antimicrobials | Concentrations of antimicrobial agents (ppm) |
| --- | --- |
| Cinnamon oil | 0, 1.56, 3.12, 6.25, 12.5 |
| ZnO NPs | 0, 6.25, 12.5, 25, 50 |
| Encapsulated curcumin | 0, 4, 8, 16, 32 |
| Cinnamon oil + 12.5 ppm ZnO NPs | 0, 1.56, 3.12, 6.25, 12.5 |

**Supplementary tables

Table S1.** Working concentrations of antimicrobial agents in time-killing method.

**Table S2.** Working concentrations of antimicrobial agents to generate concentration-effect curves.

| Antimicrobials | Concentrations of antimicrobial agents (ppm) |
| --- | --- |
| Cinnamon oil | 0, 1.87, 2.5, 3.12, 4.68, 6.25, 12.5, 25, 50 |
| ZnO NPs | 0, 1.25, 3.75, 6.25, 9.37, 16, 18, 20, 25, 30, 35, 40, 50, 100 |
| Encapsulated curcumin | 0, 2.20, 5.40, 7.2, 9, 13.50, 24, 32, 36, 40, 48, 80 |
